# Accounting for shear scission in the size-exclusion chromatography of debranched starch

**DOI:** 10.1007/s00216-025-05917-w

**Published:** 2025-05-24

**Authors:** Jihui Zhu, Ziyi Wang, Robert G. Gilbert

**Affiliations:** 1 Jiangsu Key Laboratory of Crop Genomics and Molecular Breeding, Zhongshan Biological Breeding Laboratory, Jiangsu Key Laboratory of Crop Genetics and Physiology, College of Agriculture, Yangzhou University, Yangzhou, 225009 China; 2 Centre for Nutrition and Food Sciences, Queensland Alliance for Agriculture and Food Innovation, The University of Queensland, Brisbane, QLD 4072 Australia; 3 Department of Grain Science and Industry, Kansas State University, Manhattan, KS 66506 USA

**Keywords:** Shear scission, Rice, Starch, Size-exclusion chromatography, Chain-length distribution

## Abstract

The chain-length distribution (CLD) of starch influences many functional properties of starch-containing substances, and also contains information about starch biosynthetic processes. The commonest method for measuring this CLD is to debranch the starch enzymatically, and then to measure the molecular weight distribution of the resulting linear chains using size-exclusion chromatography (SEC). However, SEC suffers from various artifacts, including shear scission of longer chains. Here, a method of correcting for such shear scission is developed: fitting the apparent CLDs (affected by shear scission) with biosynthesis-based models, over a range of flow rates, and extrapolating the resulting model-based parameters to zero flow rate. To apply this, the apparent CLDs of five rice starches were measured using SEC with a range of flow rates, and these apparent CLDs were parameterized using biosynthesis-based models. The model parameters fitted from CLDs at different flow rates were extrapolated to zero flow rate, and were then used to calculate the CLD that would be obtained with zero flow rate, thereby taking partial account of shear scission. The extrapolation suggests that shear scission significantly degrades extra-long (degree of polymerization, DP, >1500) amylose chains to shorter chains (DP ~ 500–1500), which, if uncorrected, would vitiate inferences from SEC results. The partial correction method devised here can be used to develop more reliable relationships between structural and biosynthesis-related parameters of starch and functional properties of starch-containing substances.

**Graphical Abstract:**

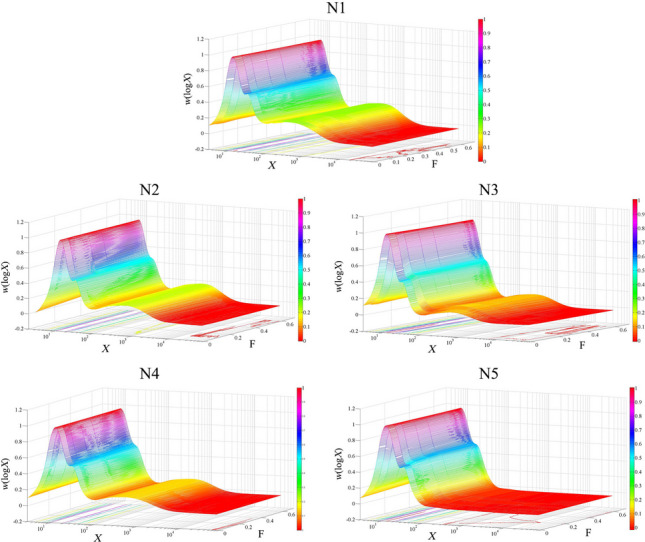

## Introduction

Starch is the major component of food energy in most diets, and it also has major industrial uses. In addition, starch is a biodegradable and renewable material. In the food industry, it is used as a thickener in food preparation and in sauces, etc. It is also used in the paper, textile, and pharmaceutical industries, in drilling, and in bioplastics. It is completely biocompatible.

Starch molecular structure controls many functional properties of the starch-containing component of the food or industrial product, such as the digestion rate in the gastrointestinal tract and thus the rate of glucose release into the bloodstream; extended high release rates can have adverse health outcomes, such as overloading the insulin response system.

Starch is a complex branched glucose polymer and, in most cases, can be divided into two components, amylopectin and amylose, both connected by (1 → 4)-α linear links and (1 → 6)-α branch points. Amylopectin is a highly branched molecule with many short-chain branches, and amylose is a largely linear long-chain molecule with a few long-chain branches [1]. Amylopectin has a high molecular weight (~10^7–8^) and large whole-molecule size, while the molecular weight of amylose is lower (~10^6–7^) and the whole-molecule size is smaller than that of amylopectin.

The structure of starch can be divided into multiple levels. The first level comprises the individual glucose chains containing (1 → 4)-α linear glycosidic linkages, and the distribution of the degrees of polymerization of individual chains is the chain-length distribution (CLD). Many studies have reported that features of the CLD correlate significantly with a number of starch-related properties of starch-containing substances, such as digestibility, palatability, viscosity, and thermal properties. Rapid digestibility is considered to relate to the increasing incidences of non-transmissible diseases, such as diabetes [2, 3], mentioned above. The second level is that of the whole starch molecules, comprising individual chains linked through (1 → 6)-α branch points. Measures of this second level include the weight and size distributions of whole starch molecules [4]. Note that for a complex branched molecule, such as starch, there is no one-to-one relation between molecular weight and molecular size distributions.

Whole-starch weight and size distributions can be measured by various techniques. Primary requirements for an ideal method are that the technique does not cause shear-induced molecular degradation of the starch, and that the dissolution process is not structurally biased, e.g., preferentially dissolving small rather than large whole molecules. Checking for a lack of degradative and size biases can be done in various ways, e.g., whether or not the apparent distribution obtained by a given technique varies with shear or with concentrations used in the abstraction. By these criteria, dissolution methods involving dimethyl sulfoxide (DMSO) appear suitable [5, 6].

Methods for size separation of starch include fluorophore-assisted carbohydrate electrophoresis (FACE), field-flow fractionation (FFF), and size-exclusion chromatography (SEC). FACE cannot be used for chains with a degree of polymerization ≳ 80 [7]. Asymmetric-flow FFF, AF^4^ (e.g., [8]), and sedimentation field-flow fractionation (sFFF) [9], have been used for this purpose; there appear to be no reports of using thermal-flow FFF in this regard. A problem is that DMSO can damage parts of the equipment normally used for this characterization, such as channels in AF^4^. While a number of reports on using FFF for degraded starch have appeared, there do not appear to be any on undegraded native starch, because of problems arising from the large size of this molecule and/or DMSO-induced damage to the equipment. Non-chromatographic methods, such as MALDI-ToF mass spectrometry, cannot be used because of the high molecular weights of native starch molecules.

Size-exclusion chromatography (SEC, which is often known as gel permeation chromatography (GPC) when employing organic solvents, or gel filtration chromatography (GFC) when employing aqueous solvents) is widely used to measure the size distribution of whole molecules [10–12]. SEC separates molecules by molecular size (but not by molecular weight, despite what is sometimes stated in textbooks), the separation parameter being defined as the SEC hydrodynamic volume (*V*_h_) [13]. It is noted that IUPAC defines this size parameter [14] from the volume of a hypothetical impenetrable sphere displaying, in a hydrodynamic field, the same frictional effect as an actual polymer molecule; this size may be different for different types of macromolecular motion, and thus may not be the same with different measurement methods, e.g., light scattering vs. SEC calibration with standards. While the “size” of a sample can vary with the measurement method, different methods are expected to preserve trends, e.g., macromolecules in a given sample being larger than those in another sample.

It is essential to be aware that there is no relation between molecular weight and molecular size for a complex branched polymer such as starch.

Quantitative SEC characterization almost always uses the universal calibration assumption [10, 13, 15] that SEC separation does not depend on the nature of the polymer but solely on *V*_h_. It is noted that this assumption has thus far only been tested in a few cases [10, 13, 15]. Using low-dispersity standards with known *R*_h_ (or well-characterized polydisperse standards [16]), one can then obtain a calibration curve of *R*_h_ against elution volume, which, however, can only be used for a particular instrument on a particular day; the nature of SEC elution is that calibration needs to be repeated on essentially a daily basis. The hydrodynamic radius, *R*_h_, as used in this study, is based on the SEC elution times compared to standards whose *R*_h_ values are obtained from dynamic light scattering, together with the universal calibration assumption; this is a commonly used definition.

To analyze chain-length distributions using SEC, the first step is to break each branch point in the starch sample by enzymatic debranching, whereafter the weight distribution *w*(log*R*_h_) can be obtained as above by using a mass-sensitive detector. For the individual linear chains obtained by debranching amylose and amylopectin, there are one-to-one relations between the molecular weight, molecular size, and the degree of polymerization (DP) *X* [17], which means that a given *w*(log*R*_h_) can be converted to the weight distribution of *X*, *w*(log*X*). SEC has, however, the drawback of the occurrence of significant chain scission through shear degradation in the system “plumbing” [18–22]. This shear degradation may be caused by both shear forces and extensional forces; for convenience, we use “shear scission” to refer to scission by either or both of these. Dimensionless analysis shows that this artifact cannot be avoided for current or foreseeable SEC systems [22]. Partially correcting for this artifact is the topic of the present paper.

Other techniques, such as fluorophore-assisted carbohydrate electrophoresis (FACE [23]), can be used to give the number distribution of linear starch chains, but only for shorter chains. SEC has essentially no upper limit for chain length. For linear polymers, the weight and number distributions are related by [17]:

$$w\left(\text{log}X\right)={X}^{2}{N}_{de}\left(X\right),$$

where *N*_de_(*X*) is the number of chains with DP *X* after debranching.

An important requirement for making use of such data is to be able to express the resulting distribution functions, *N*_de_(*X*) or *w*(log*X*), in terms of appropriate models with a small number of parameters; these parameters can then be used subsequently for obtaining biosynthesis-structure-property relations. For this purpose, two biosynthesis-based models have been developed, one for amylopectin [24] and another for amylose [25]. These both involve the assumption that CLD curves represent contributions from a number of “enzyme sets”. Each set comprises one starch synthase, one or two starch branching enzymes, and one debranching enzyme. The fitting procedure reduces all the enzymatic activities to two parameters, *β* and *h*, for each set; *β* is the ratio of the activity of the starch branching enzyme to that of the starch synthase in that set, and *h* is the relative contribution of each set to the overall CLD. Each enzyme set is the major, but not sole, contributor to the CLD over a chosen range of DP; i.e., the DP ranges overlap for different sets. To fit CLD data, one first least-squares fits the data to the models for each chosen DP range to give a first estimate of the values of *β* and *h* for each set. The whole CLD is then globally least-squares fitted to the sum of the contributions from each set to provide *β* and *h* values for each of the sets. This global fitting ensures that the final result is not sensitive to the initial choice of DP range for each set.

Flow rate is considered one of the significant factors that causes shear scission, other factors including the diameter of the column packing particles, the cross-sectional area of the column, and the column porosity; flow rate is the most easily varied. The present study puts forward the following way of at least partially accounting for this artifact for starch chains, based on the aforementioned models. Five starch samples are used. CLDs are measured by SEC for these starches under five different flow rates (0.6, 0.5, 0.3, 0.2, 0.1, and 0.05 mL/min). The biosynthesis-based models are then fitted to the resulting CLDs, noting that although these are not the true CLDs because of shear scission, nevertheless the functional forms from the models still provide a good fit to the data. The values of *β* and *h* so obtained for each region and flow rate are then extrapolated back to zero flow rate, so as to at least partially account for shear scission. The values for *β* and *h* obtained by these extrapolations are then used in the models to generate CLDs corrected for shear scission.

It is important to be aware that while the methodology developed here can be generally applied to any SEC set-up to correct for shear scission, the specific parameters we obtain in this paper are only applicable to the particular SEC system used here.

## Materials and methods

Flow rate is considered one of the significant factors that cause shear scission, other factors including the diameter of the column packing particles, the cross-sectional area of the column, and the column porosity; flow rate is the most easily varied.

### Materials

Five rice varieties (N1–N5, Table 1) were purchased from food markets in Brisbane, Queensland, Australia. All samples were popular varieties in local shops, were grown in four different countries, and can be divided into different rice subpopulations. These varieties are suitable to explore shear scission, as they show a wide diversity of starch fine structure and amylose content, including one glutinous rice that contains only ~ 1.24% amylose.

**Table 1:** Rice varieties used in this study

ID	Name of species	Country of growth	Sub-population	Amylose content %
N1	BASMATI	India	Aromatic	28.4
N2	LOW GI	Australia	N	29.2
N3	AKITAKOMACHI	Japan	Japonica	17.3
N4	KOSHIHIKARI	Japan	Japonica	21.6
N5	DRAGON	Laos	Japonica	1.24

### Starch extraction and debranching

Rice white grains (~1 g) were hand-ground into flour and filtered through a 100-mesh and then a 200-mesh sieve. The meshed flour was then mixed with 25 mL protease solution (Megazyme, Wicklow, Co. Bray, Ireland; 50 mg, ∼20,000 U/g) containing sodium nitrate (0.04 g/mL) and then kept for 16 h at 42℃ to remove proteins. The resulting mixture was then centrifuged, and the supernatant was discarded. The residue was washed three times using deionized water to remove any remaining amino acids, then washed twice with absolute ethanol. The final residue (purified starch) was precipitated by centrifugation (4000 ×*g* for 10 min) and freeze-dried for 12 h.

For CLD characterization, 7∼9 mg purified rice starch was measured and dissolved in 1.5 mL dimethyl sulfoxide (DMSO) containing 0.5% LiBr (w/w) for 12 h with gentle agitation; starch was then reprecipitated by adding 8 mL absolute ethanol and centrifuging (4000 ×*g*, 10 min) with the supernatant being discarded. The precipitate was then washed three times using 8 mL of absolute ethanol to remove the remaining DMSO. The residue was then suspended in 0.9 mL of warm distilled water and kept in a boiling water bath for 15~30 min until the starch was fully gelatinized. The solution was then cooled to room temperature and mixed with 1 mL of acetate buffer (0.1 M, pH ∼3.5) containing sodium azide (0.04 g/mL) and isoamylase (2.5 mL, Megazyme). After incubating the mixture in a water bath at 37 °C for 3 h, the pH was neutralized to ~7 by adding sodium hydroxide solution (0.1 M), followed by incubating at 80 °C for 1 h, and then freeze-dried for 12 h. The dried debranched starch was re-dissolved in DMSO/LiBr (4−6 mg/mL), kept at 80 °C for 24 h, then centrifuged (4000 ×*g*, 10 min), and the supernatant was then transferred to SEC phials for further analysis by SEC.

### Measuring and fitting the chain-length distribution

Size-exclusion chromatography separates polymer molecules by a measure of molecular size, termed the hydrodynamic radius *R*_h_. Using the one-to-one relations between molecular weight, molecular size, and the degree of polymerization (DP) *X* of linear (e.g., debranched) homopolymers, SEC with a mass-sensitive detector (e.g., a differential refractive index, DRI, detector, as used here) gives the SEC weight distribution of chains, *w*(log*X*). Relatively monodisperse pullulan standards (Polymer Standards Services, Mainz, Germany) with varying molecular weights from 180 Da to 1.22 × 10^6^ Da, dissolved in DMSO/LiBr eluent (DMSO, 0.5% LiBr, 80 °C), were used for calibration. A Shimadzu SEC (Shimadzu LC-20 AD Prominence Liquid Chromatograph) was used to implement starch separation, with a degassing unit (Shimadzu DGU-20 A3R), an auto-sampler (Shimadzu SIL-20 AC HT), a communication bus module (Shimadzu CBM-20 A), and a refractive index detector (Shimadzu RID-20 A). The columns used were 8 × 50 mm, 10 μm, GRAM Pre-column (Agilent, Santa Clara, CA, USA), and 8 × 300 mm, 10 μm GRAM 100 and 8 × 300 mm, 10 μm GRAM 1000 analytical columns (Agilent, Santa Clara, CA, USA), encased in a column oven (Shimadzu CTO-20 A) at 80 °C. The sampling solution concentration was 4 mg/mL, the injection volume was 100 µL, and the flow rates were set at 0.05, 0.1, 0.2, 0.3, 0.5, and 0.6 mL/min.

A common method to extract biological information about CLD involves empirically defining CLD regions and obtaining the relative number of chains in each region. However, different choices of regions might significantly affect the inferences [26, 27]. Moreover, SEC suffers from band broadening, which can significantly alter the shape of a distribution, especially at the lowest flow rates used here [22, 28, 29]. We here reduce such problems by fitting the CLDs to two biosynthesis-based models [24, 25, 30], where the fitting partially takes band broadening into account; this fitting is not empirical.

The models, one each for amylopectin and amylose, assume that the CLD is comprised of contributions from a number (typically three) of enzyme sets, each set dominating a given DP range, while allowing other enzyme sets to be lesser contributors to that range. The biosynthesis of starch is dominated by the isoforms of three major enzyme types, namely starch synthases, SSs (including granule-bound starch synthase, GBSSI), which cause chain growth, while starch branching enzymes (SBEs) and debranching enzymes (DBEs) are the chain-stoppage events. The model parameters in a given enzyme set *i* comprise the values of *β*_*i*_ and *h*_*i*_ for each set *i*; *β*_*i*_ is the ratio of the activity of the dominant starch branching enzyme(s) to that of the dominant starch synthase in that DP range, and *h*_*i*_ is the relative contribution of that enzyme set to the overall CLD. In brief, a larger value of *h* in a given DP region implies relatively more chains in that region, and a larger value of *β* means more shorter chains in that region [26].

The first step in the fitting procedure starts with initially assuming a single enzyme set is the sole contributor to a given CLD range, to obtain a first estimate of the fitting parameters by a least-squares fit of the model over this range, and then refining the resulting values of the fitting parameters by a global least-squares fit of all parameter values over the entire DP range. This way, the final values of the fitted parameters do not depend on the choice of DP range for the initial fit used to obtain first estimates of the fitting parameters. Computer codes for fitting these models are available for public download without cost [24, 25].

Fitting CLDs to these models yields nonempirical, biologically meaningful parameters, *β*_*i*_ and *h*_*i*_, representing the features of different CLD regions; these parameters can be used with the mathematical models to generate the entire starch CLD and can also be used to find correlations with starch properties of interest.

### Statistical analysis

SPSS 22.0 (Statistical Graphics Corp., Princeton, NJ, USA) is used here to obtain Pearson correlations. Correlations with probability values of *p* < 0.01 and *p* < 0.05 are considered statistically very significant and significant, respectively. MATLAB (MathWorks, Natick, MA, USA) is used to extrapolate CLDs and their features under different flow rates using the functions of *interp1* and *griddedInterpolant*; the linear method is chosen for both interpolation and extrapolation.

## Results and discussion

### Effects of SEC shear scission on CLDs

**Fig. 1:**
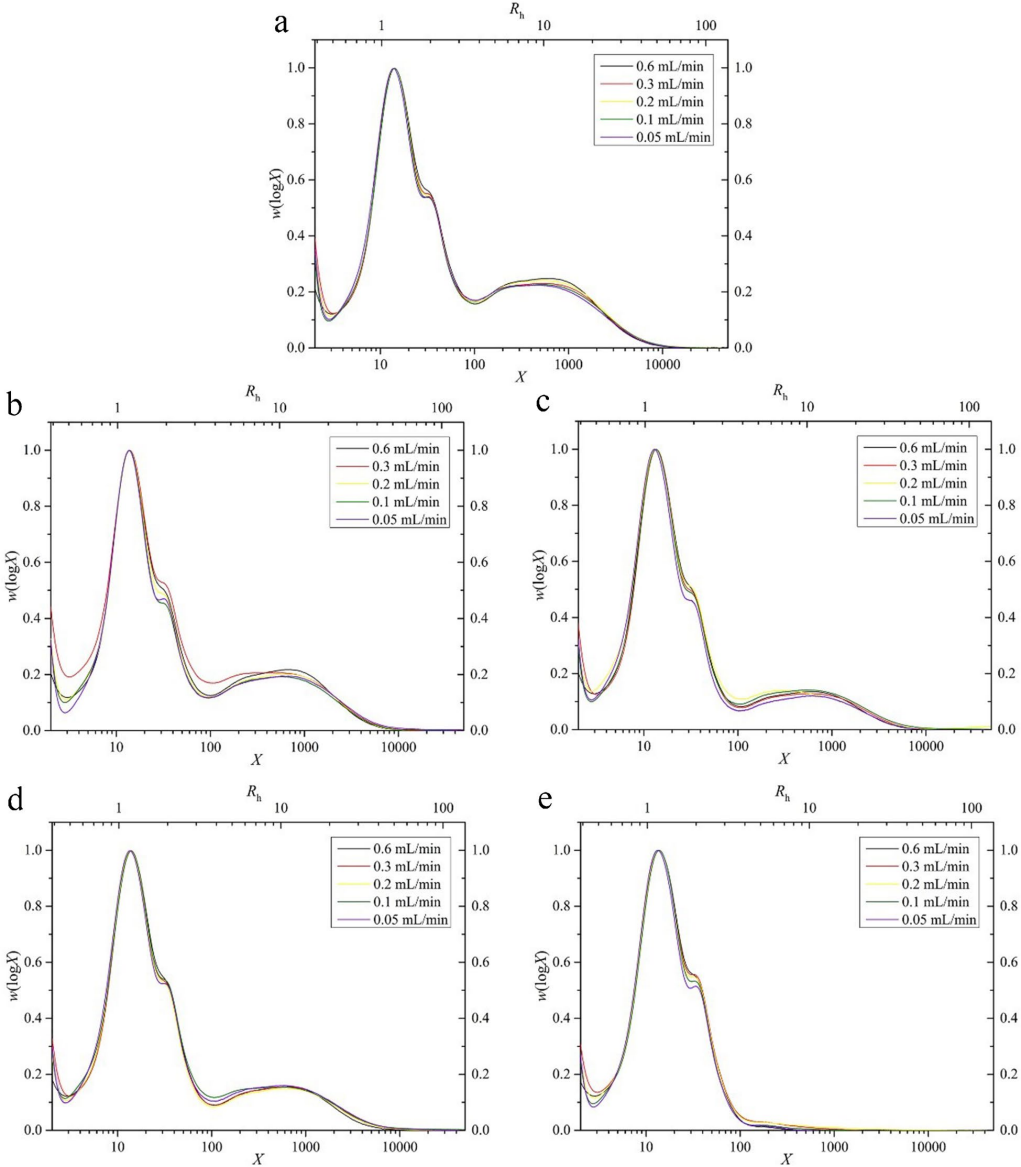
SEC weight CLDs, *w*(log*X*), as functions of the degree of polymerization *X*, of debranched starch for the five rice varieties: **a**, N1; **b**, N2; **c**, N3; **d**, N4; and **e**, N5, under five flow rates, 0.6, 0.3, 0.2, 0.1, and 0.05 mL/min

Examination of the effects of shear scission on the enzymatically debranched starches was carried out using five SEC flow rates (0.6, 0.3, 0.2, 0.1, and 0.05 mL/min). The apparent SEC weight distributions so obtained are shown in Fig. 1. Chains with DPs *<* 100 (*R*_h_ ≲ 3.7 nm) are amylopectin chains, and those with *X* over 100 are amylose chains. Figure 1a–d shows the chain-length distributions of those varieties, N1–N4, which contain both amylopectin and amylose, while Fig. 1e is for the glutinous rice starch (variety N5) with amylose content ~1.24%. The CLDs are similar to those seen in many other studies, e.g., [31].

As is commonly seen, there are two regions in the CLDs of amylopectin chains in all five varieties. The first region, with DP < 20, comprises chains occupying only a single crystalline lamella, and the second region, with DP ≳ 20, comprises chains crossing more than one lamella. There are no obvious differences in the CLDs of the first region for different flow rates, while clear differences can be observed in the second region. For example, the CLD at the fastest flow rate (0.6 mL/min) for variety N1 has the highest value of the second peak, and the heights of the second peak decrease with decreasing flow rates (Fig. 1a). Similar trends are found in the other four varieties (N2–N5). This indicates that a faster flow rate is correlated to more intermediate amylopectin chains, showing that, as expected, a fast flow rate shears longer chains into shorter ones and thus causes a relative apparent increase of the amount of intermediate amylopectin chains. Moreover, significant differences can be seen in the apparent distributions for longer amylopectin chains, in the DP range 60~100 (Fig. 1), implying that SEC shear scission affects the amounts of long amylopectin chains, as expected.

As usual in such cases, two peaks in the amylose CLDs are seen in Fig. 1. The first peak at DP ~200 belongs to the short amylose chains, and the other at DP ~1000 is in the long amylose chain region. As seen in Fig. 1a–c (N1–N3), a faster flow rate is correlated to a higher value of both amylose peaks in the CLDs. Thus, a fast flow rate can contribute to the apparent appearance of relatively more amylose short and long chains, indicating that these two regions are significantly affected by SEC shear scission.

For all four amylose CLDs (Fig. 1a–d, N1–N4), the extra-long amylose chains (DP ≳ ~1500) suffer the greatest SEC shear scission, as seen previously [22]. That is, a faster flow rate shears more amylose molecules, and thus reduces the amount of extra-long amylose chains.

### Fitting CLDs to biosynthesis models

**Fig. 2:**
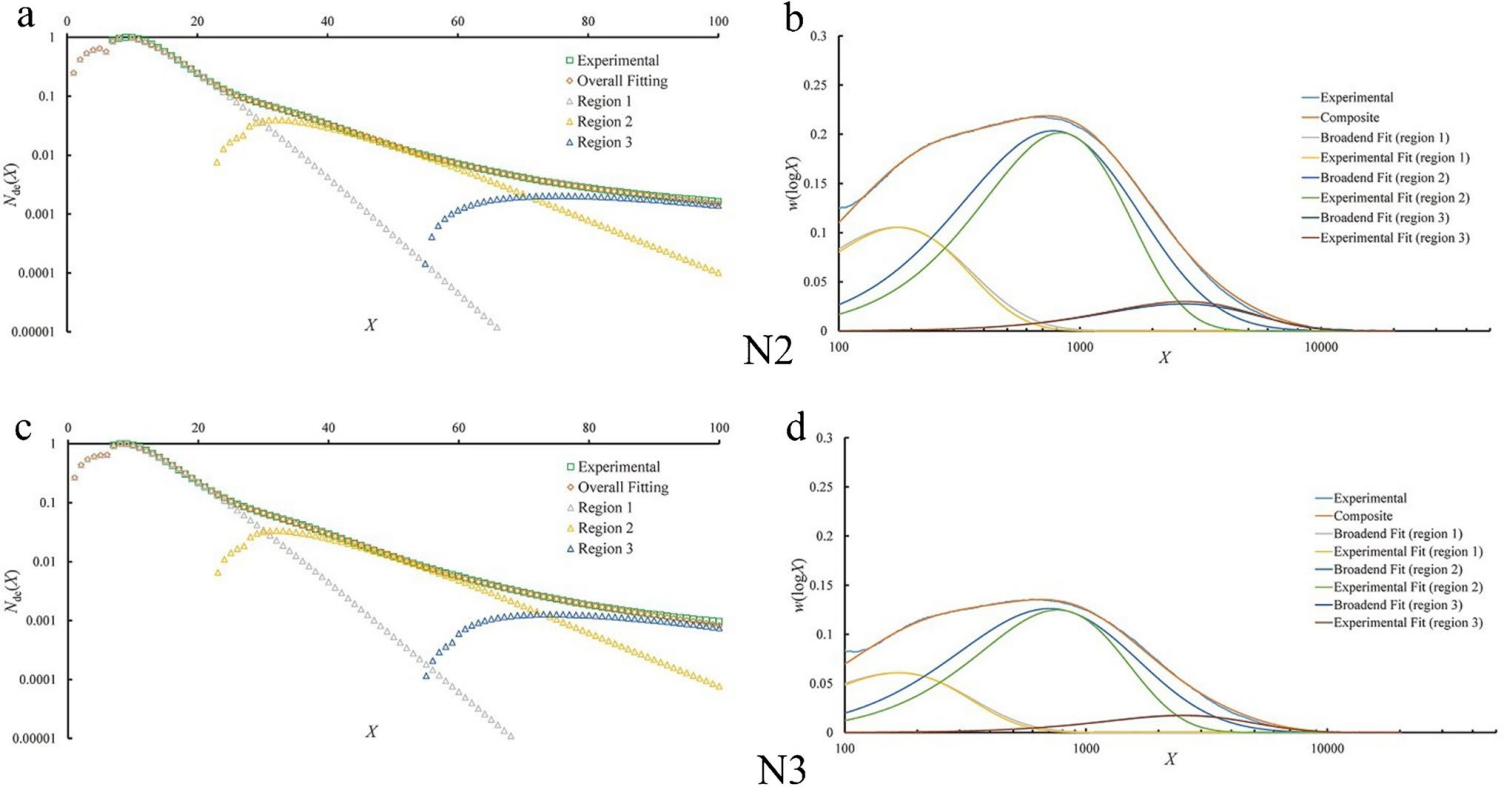
Fitting results for amylopectin and amylose CLDs for N2 (LOW GI) and N3 (AKITAKOMACHI). Amylopectin CLD fitting (**a**) and (**c**), and amylose CLD fitting (**b**) and (**d**)

The two biosynthesis-based models mentioned above were used to fit the CLDs; two typical fitted results are shown in Fig. 2. With the 0.6 mL/min flow rate, both N2 and N3 show more long amylose chains (DP 500–1500) than short (DP 100–500) and extra-long (DP > 1500) amylose chains, while the amount of short and long amylose chains of N2 is more than that of N3. Thus, varieties with different amounts of amylose chains show similar trends. The values of the parameters of all experimental CLDs are given in Table 2.

**Table 2:** Starch structural parameters obtained from SEC weight CLDs of debranched starches under five flow rates, and the parameters interpolated or extrapolated from experimental data. *V*, sample name; *FR*, flow rate (mL/min); *S*, sample source

V	FR	S	*h*_Ap,1_	*h*_Ap,3_×10^2^	*h*_Ap,5_×10^3^	*β*_Ap,1_×10	*β*_Ap,3_×10^2^	*β*_Ap,5_×10^2^	*h*_Am,1_×10^2^	*h*_Am,2_×10^2^	*h*_Am,3_×10^2^	*β*_Am,1_×10^2^	*β*_Am,2_×10^3^	*β*_Am,3_×10^4^
			Amylopectin CLDs						Amylose CLDs					
N1	0.6	A	0.991	4.23	2.47	1.76	5.75	2.24	10.11	1.82	3.65	1.20	2.55	6.8
	0.5	B	0.994	4.27	2.54	1.76	5.84	2.30	9.70	1.78	3.68	1.22	2.59	6.7
	0.4	B	0.997	4.31	2.61	1.77	5.93	2.37	9.30	1.74	3.70	1.24	2.64	6.5
	0.3	A	1.000	4.35	2.68	1.77	6.02	2.43	8.89	1.70	3.73	1.26	2.69	6.3
	0.2	A	1.005	4.22	2.55	1.79	5.82	2.17	10.36	1.73	3.81	1.17	2.46	6.2
	0.1	A	1.011	4.23	2.47	1.79	5.89	2.16	9.77	1.61	3.96	1.22	2.55	6.3
	0.05	A	0.993	4.21	2.60	1.87	5.99	2.31	10.44	1.56	4.28	1.27	2.69	7.0
	0	C	0.975	4.20	2.72	1.94	6.10	2.46	11.11	1.50	4.60	1.32	2.83	7.7
		D	0.75	1.21	3.11	2.13	1.70	4.53	5.68	5.55	5.73	3.01	3.48	5.11
N2	0.6	A	1.016	3.97	2.04	1.84	6.13	2.31	8.42	1.61	2.47	1.13	2.41	7.4
	0.5	B	1.013	4.05	2.05	1.93	6.22	2.33	8.31	1.57	2.69	1.13	2.43	7.5
	0.4	B	1.009	4.13	2.05	2.02	6.31	2.34	8.19	1.53	2.92	1.13	2.45	7.6
	0.3	B	1.006	4.22	2.05	2.11	6.4	2.36	8.08	1.49	3.14	1.13	2.47	7.7
	0.2	A	1.002	4.30	2.06	2.19	6.49	2.38	7.96	1.45	3.37	1.13	2.49	7.8
	0.1	A	1.011	4.04	1.96	2.26	6.31	2.42	7.38	1.36	3.54	1.16	2.64	7.6
	0.05	A	1.010	4.28	2.03	2.29	6.70	2.44	7.10	1.38	4.08	1.20	2.69	7.2
	0	C	1.009	4.52	2.10	2.31	7.09	2.46	6.82	1.40	4.62	1.25	2.74	6.9
		D	0.75	3.46	9.19	9.50	5.96	5.57	12.30	6.03	16.02	5.16	14.09	3.67
N3	0.6	A	0.997	3.35	1.28	1.64	6.23	2.63	4.88	1.00	1.44	1.19	2.63	8.0
	0.5	B	1.002	3.38	1.26	1.63	6.23	2.57	4.80	0.98	1.50	1.18	2.60	7.7
	0.4	B	1.007	3.40	1.23	1.63	6.23	2.51	4.71	0.96	1.57	1.16	2.58	7.4
	0.3	A	1.012	3.43	1.21	1.63	6.23	2.46	4.63	0.94	1.63	1.15	2.56	7.2
	0.2	B	1.016	3.43	1.29	1.65	6.28	2.42	5.14	0.98	1.81	1.20	2.56	6.7
	0.1	A	1.020	3.42	1.38	1.66	6.33	2.39	5.64	1.02	1.99	1.24	2.57	6.3
	0.05	A	0.999	3.25	1.08	1.76	7.06	2.69	4.23	0.80	3.41	1.21	2.96	9.7
	0	C	0.979	3.07	0.78	1.86	7.80	3.00	2.81	0.58	4.83	1.17	3.34	13.2
		D	1.18	2.00	13.05	2.90	5.28	5.13	17.71	8.79	34.58	2.62	5.47	15.03
N4	0.6	A	1.002	3.64	1.41	1.57	6.13	2.48	5.47	1.19	0.66	1.12	2.45	5.4
	0.5	B	1.006	3.66	1.42	1.58	6.14	2.46	5.36	1.18	0.95	1.13	2.47	5.6
	0.4	B	1.010	3.67	1.43	1.58	6.16	2.43	5.25	1.17	1.25	1.14	2.49	5.8
	0.3	A	1.014	3.69	1.43	1.58	6.18	2.40	5.15	1.16	1.54	1.15	2.51	6.0
	0.2	A	1.014	3.73	1.37	1.61	6.42	2.46	4.95	1.12	1.51	1.16	2.51	6.1
	0.1	B	1.004	3.72	1.55	1.64	6.53	2.49	5.54	1.15	1.82	1.20	2.55	6.0
	0.05	A	0.999	3.71	1.64	1.66	6.59	2.50	5.84	1.16	1.98	1.22	2.57	6.0
	0	C	0.994	3.70	1.73	1.67	6.65	2.51	6.13	1.18	2.14	1.24	2.60	6.0
		D	0.66	0.93	10.78	1.92	3.31	3.76	10.56	2.15	29.98	6.12	1.80	4.73
N5	0.6	A	0.977	3.74	0.95	1.57	6.21	3.52	-	-	-	-	-	-
	0.5	B	0.978	3.76	1.00	1.58	6.22	3.43	-	-	-	-	-	-
	0.4	B	0.979	3.79	1.05	1.59	6.22	3.35	-	-	-	-	-	-
	0.3	A	0.981	3.82	1.10	1.59	6.23	3.26	-	-	-	-	-	-
	0.2	A	0.985	3.76	1.05	1.60	6.27	3.24	-	-	-	-	-	-
	0.1	A	0.998	3.79	0.94	1.59	6.64	3.57	-	-	-	-	-	-
	0.05	A	0.984	3.63	0.94	1.67	6.76	3.65	-	-	-	-	-	-
	0	C	0.970	3.47	0.94	1.74	6.88	3.73	-	-	-	-	-	-
		D	0.73	1.71	6.70	2.01	3.59	4.82	-	-	-	-	-	-

*V*, varieties; *FR*, flow rate; *S*, source; *A*, experimental data; *B*, interpolated data; *C*, extrapolated data; *D*, coefficient of variation% of experimental data

Fitting the amylopectin CLDs to the model gives six parameters: *h*_ap,*i*_ and *β*_ap,*i*_, where the values of the subscript *i* = 1, 3, and 5 indicate short (DP 7–22), intermediate (DP 28–54), and long (DP 68–86) amylopectin chains, respectively. The fitted parameters are shown in Table 2. The values of *h*_ap,1_ are far larger than those of *h*_ap,3_, and *h*_ap,5_ shows the lowest value, indicating that amylopectin CLDs have the greatest relative amount of short chains and a lesser relative amount of long chains, as seen elsewhere, e.g., [32].

Six parameters (*h*_am,*j*_ and *β*_am,*j*_) can be obtained by fitting the amylose CLDs to the appropriate biosynthesis-based model. Here, *j* = 1, 2, and 3 represent the short (DP 100–500), long (DP 5–1500), and extra-long (DP > 1500) amylose regions, respectively. Based on the fitting results seen in Table 2, it is seen that although four whole-starch varieties (N1–N4) have different amylose contents, they have the largest values of *h*_am,2_ and the smallest values of *h*_am,3_. For example, the values of *h*_am,2_ (0.6 mL/min flow rate) of N1 and N2 are 182 and 161, respectively, while the corresponding *h*_am,3_ values are 36.5 and 24.7, showing that these varieties have the largest relative amounts of amylose long chains and the lowest relative amounts of extra-long amylose chains.

Table 2 shows the relative coefficients of variation (CV%) of five varieties under the various flow rates. The CV% values of *h*_am,1_, *h*_am,2_, and *h*_am,3_ (5.68, 5.55, and 5.73, respectively) are relatively larger than those of *h*_ap,1_, *h*_ap,3_, and *h*_ap,5_ (0.75, 1.21, and 3.11, respectively), while the largest *h* value is *h*_am,3_, 5.73. Moreover, for N1, a similar trend is seen in the CV% value of *β*, with the largest value being for *β*_am,3_, 5.11. This shows the unsurprising results that amylose chains are more strongly affected by the flow rate, which significantly correlates to SEC shear scission, than is the case with amylopectin chains, and that the extra-long amylose chain region is most affected among all CLD regions. For N2, N3, and N4, it is similarly found that *h*_am,3_ has the highest CV% values: 16.0, 34.6, and 30.0, respectively. In addition, it is found that in all five samples, the CV% of *h*_ap,5_ is significantly higher than that of *h*_ap,1_ and *h*_ap,3_. This shows that shear scission significantly affects the amylopectin long-chain region: for example, long amylose chains undergo shear scission into shorter chains, which elute in the amylopectin region.

### Correlations between starch structural features and flow rate

The correlations between flow rates and twelve CLD structural parameters were calculated by SPSS 20.0 (Statistical Graphics Corp., Princeton, NJ), and results are shown in Table 3. For varieties N1 and N2, two parameters, namely *h*_am,2_ and *h*_am,3_, are significantly positively correlated (correlation coefficients 0.921* and 0.986*, where the single asterisk denotes statistically significant) and negatively (−0.819* and −0.962**, where the double asterisk denotes highly statistically significant) correlated to flow rate, respectively. This indicates that long and extra-long amylose chain regions are significantly affected by flow rate in this system, in which, with the increasing of the flow rate, the amount of extra-long amylose chains decreases while the amount of long amylose chains increases. This implies that, in this system, the region of extra-long amylose chains suffers most from SEC shear scission, and the large molecular extra-long amylose chains are more prone to be sheared to smaller molecules; both results are unsurprising. In addition, *h*_am,2_ and *h*_am,3_ show moderately high correlation coefficients with flow rate for variety N3: 0.522 and −0.700, respectively.

**Table 3:** Correlations between flow rates and fitted parameters

S	*h*_Ap,1_	*h*_Ap,3_	*h*_Ap,5_	*β*_Ap,1_	*β*_Ap,3_	*β*_Ap,5_	*h*_Am,1_	*h*_Am,2_	*h*_Am,3_	*β*_Am,1_	*β*_Am,2_	*β*_Am,3_
N1	−0.518	0.176	−0.229	−0.743	−0.600	0.103	−0.163	0.921*	−0.819*	−0.310	−0.199	0.138
N2	0.212	−0.641	0.196	−0.876	−0.507	−0.461	0.532	0.986**	−0.962**	−0.182	−0.108	−0.187
N3	−0.235	0.187	0.039	−0.685	−0.543	0.224	−0.105	0.522	−0.700	−0.379	−0.456	−0.087
N4	−0.174	−0.892*	−0.639	−0.675	−0.441	0.325	−0.408	0.695	−0.974**	−0.719	−0.954*	−0.494
N5	−0.699	0.249	0.075	−0.696	−0.781	−0.205						

*Correlation is significant at the 0.05 level; **correlation is significant at the 0.01 level

For variety N4, three parameters were found to be significantly correlated to flow rate: *h*_am,3_ (−0.947**), *β*_am,2_ (−0.954*), and *h*_ap,3_ (−0.892*). This shows that the apparent amount of extra-long chains decreases with increasing flow rate, similar to what is seen in N1 and N2. However, it was found that the amount of long amylose chains is not significantly increased with flow rate (0.522), as seen in N1 and N2; this might be because the shorter chains (DP 500~1000, *β*_am,2_, −0.954*) in the region of long amylose chains are significantly sheared, suggesting that smaller molecules, such as DP 500~1000 amylose chains, are sheared in the SEC set-up used here. Furthermore, it is found that the decrease in flow rate is significantly correlated (*h*_ap,3_, −0.892*) to the increasing amount of intermediate amylopectin chains (DP ≈ 28~54) in N4; there is a possibility that the slower flow rate has an accumulation effect on small molecules, and thus more smaller molecules are detected under a slow flow rate. Furthermore, even though a study has reported that shear scission of a large polymer is most likely to produce two chains of approximately the same size [33], it is still possible that significant numbers of long chains are snipped to non-equal length chains.

In addition to the significant correlation with flow rate (*h*_ap,3_, −0.892*) found in N4, high but not significant negative correlations have been found between *β*_ap,1_ and flow rates of all five samples (correlation coefficients 0.743, −0.876, −0.685, −0.675, and −0.696, respectively) and between *β*_ap,3_ and flow rate. It appears that reducing the flow rate causes more shorter chains in the short (DP 7–22) and intermediate (DP 28–54) amylopectin chain regions, probably due to the slower flow rate triggering the accumulation of longer chains from short (DP 7–22) and intermediate (DP 28–54) amylopectin regions; therefore, the relative amount of shorter chains in these two regions actually increases.

### Accounting for SEC shear scission

Amylose long and extra-long chains are found here to be significantly affected by flow rate (Table 3), indicating that a faster flow rate leads to a lower relative amount of amylose extra-long chains and a higher relative amount of amylose long chains: a faster SEC flow rate breaks the extra-long amylose chains into amylose long chains and/or shorter amylose chains. It is noted that the extra-long amylose chain scission (random degradation of polymer chains) happens when linear macromolecules (such as debranched amylose chains) are exposed to transient extensional flows. Obviously, a slower flow rate reduces the shear scission effect, and the shear scission can, in principle, be eliminated under zero flow rate. However, it is impossible to run SEC under zero flow rate; moreover, SEC band broadening increases with lower flow rates, meaning lower resolution. The extrapolation and interpolation method used here enables the CLD to be predicted, which would be obtained under a hypothetical zero flow rate, this being unobtainable in practice.

The interpolation and extrapolation were implemented using MatLab (MathWorks, Natick, MA, USA), with the function *griddedInterpolant*, and the predicted starch structural parameters under flow rates 0.5, 0.4, and 0 are listed in Table 2, S (source) B, and C, respectively. It is found that *h*_am,3_ shows the highest values in samples N1, N2, N3, and N4 (46.0, 46.2, 48.3, and 21.4) under the predicted hypothetical zero flow rate. This indicated that the amount of amylose extra-long chains is increased under a slower flow rate, and the amount of amylose long chains is increased due to the fact that *h*_am,2_ shows the lowest value in samples N1 and N3 (150.2 and 58.1) and relatively lower values in samples N2 and N4 (134.0 and 117.7), respectively.

**Fig. 3:**
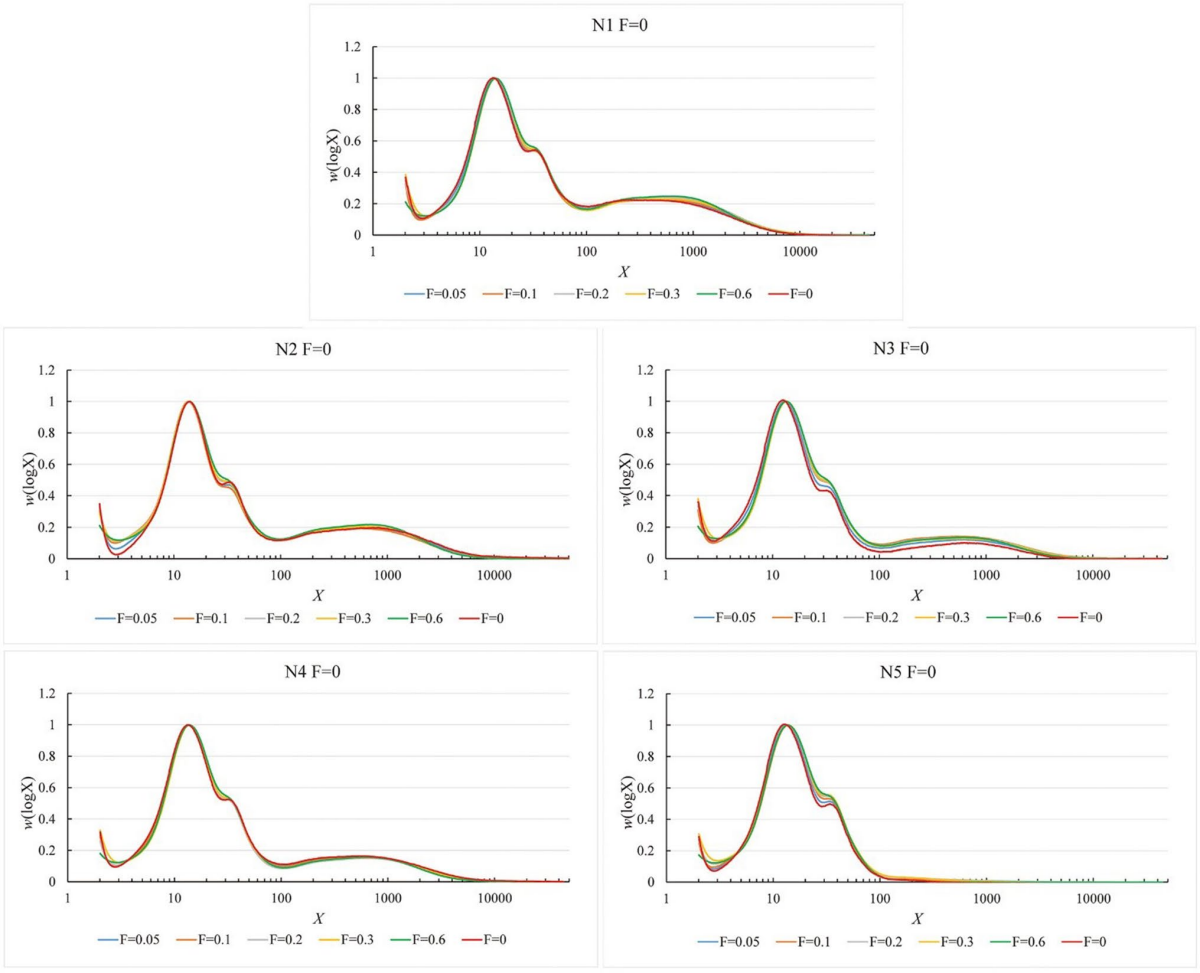
The weight CLDs of debranched starch of five rice varieties under five flow rates, and the hypothetical result by extrapolating flow rate to zero. F, flow rate (mL/min)

**Fig. 4:**
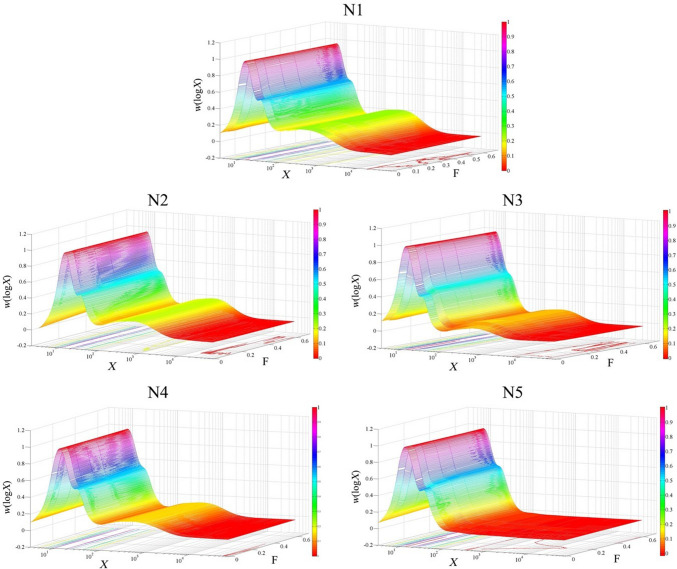
3D plots of the weight CLDs of debranched starch of five rice varieties under five flow rates, and extrapolation of flow rate to zero. F, flow rate (mL/min)

CLDs under a hypothetical zero flow rate were calculated using the Matlab function *interp1* (Fig. 3). It is found that the amounts of extra-long amylose chains of samples N2 and N4 are higher under the zero flow rate. In addition, the amounts of chains between the CLD range DP 20–30 are reduced under the zero flow rate; this suggests that the accumulated effect on small molecules is able to modify the CLDs, or the extra-long amylose chains can possibly be sheared to shorter chains, such as to DP 20–30, and thus increase the apparent relative amounts of such chains in a given region under a faster flow rate. This assumption is supported by the significant negative correlation (–0.89*) found between *h*_ap,3_ and flow rate in N4, and the high negative correlations between *β*_ap,1_ and flow rates in all five samples. The extrapolation of CLDs under the zero flow rate is displayed in 3D in Fig. 4.

## Conclusion

SEC shear scission has a significant effect on the apparent size distributions of large starch molecules, particularly for whole molecules of amylopectin and chains of debranched amylose [22, 32]. The present paper develops a means of partially correcting for this for debranched chains. Five flow rates (0.05–0.6 mL/min) were used to measure the apparent CLDs of five rice samples; the CLDs were then fitted by starch biosynthesis-based models to obtain starch structural parameters for the CLDs at different flow rates. These structural parameters were then extrapolated to zero flow rate, to account, at least partially, for shear-scission artifacts in the apparent SEC distributions.

The correlations between starch structural parameters and flow rates show that extra-long amylose chain region suffers most from SEC shear scission, and, due to the large molecular size, extra-long amylose chains are degraded to shorter chains, appearing in the SEC elution region appropriate to long amylose chains. Furthermore, extra-long amylose chains can also be snipped into shorter chains, eluting in the SEC region expected for debranched amylopectin; the effects on CLDs under different flow rates are seen in Fig. 3.

SEC flow rate is a major factor causing shear scission of larger chains. Although the size distribution of larger chains that are sheared in SEC can be obtained by using alternative size-separation methods, such as field-flow fractionation [8, 34–39], these methods also suffer from shear scission (although less so than in SEC). Decreasing flow rate reduces the shear scission of smaller chains.

Here, an extrapolation means of partially accounting for the effects of shear scission has been developed. This can help breeders and grain selectors obtain the true chain-length distributions of debranched starch, building more accurate relationships between starch fine structure and other properties, such as digestibility and pasting characteristics, which can be useful in rice variety selection and breeding, as these distributions are important determinants of functional properties. In addition, although the CLD in this study is similar to those reported elsewhere, in the future one could obtain new mechanistic information by using polymers with “tunable” branching, for example, obtained by one of the various types of controlled radical polymerization (although it is hard to use this method to obtain high molecular weights).

## Data Availability

Data are available from the authors on request.

## References

[1] Takeda Y, Shitaozono T, Hizukuri S. Structures of sub-fractions of corn amylose. Carbohydr Res. 1990;199:207–14. 10.1016/0008-6215(90)84262-S.

[2] Zhu J, Bai Y, Gilbert RG. Effects of the molecular structure of starch in foods on human health. Foods. 2023;12:2263. 10.3390/foods12112263. 37297507 10.3390/foods12112263 PMC10252388

[3] Seal CJ, Daly ME, Thomas LC, Bal W, Birkett AM, Jeffcoat R, Mathers JC. Postprandial carbohydrate metabolism in healthy subjects and those with type 2 diabetes fed starches with slow and rapid hydrolysis rates determined in vitro. Br J Nutr. 2003;90(5):853–64. 10.1079/BJN2003972. 14667179 10.1079/bjn2003972

[4] Li C, Yu W, Gilbert RG. The effects of starch molecular fine structure on thermal and digestion properties of rice starch. Foods. 2022;11:4012. 10.3390/foods11244012. 36553754 10.3390/foods11244012 PMC9778140

[5] Dona A, Yuen C-WW, Peate J, Gilbert RG, Castignolles P, Gaborieau M. A new NMR method for directly monitoring and quantifying the dissolution kinetics of starch in DMSO. Carbohydr Res. 2007;342:2604–10. 10.1016/j.carres.2007.08.010. 17892866 10.1016/j.carres.2007.08.010

[6] Schmitz S, Dona AC, Castignolles P, Gilbert RG, Gaborieau M. Quantification of the extent of starch dissolution in dimethylsulfoxide by 1H NMR spectroscopy. Macromol Biosci. 2009;9(5):506–14. 10.1002/mabi.200800244. 19089874 10.1002/mabi.200800244

[7] Wu AC, Li E, Gilbert RG. Exploring extraction/dissolution procedures for analysis of starch chain-length distributions. Carbohydr Polym. 2014;114(1):36–42. 10.1016/j.carbpol.2014.08.001. 25263861 10.1016/j.carbpol.2014.08.001

[8] Rojas CC, Wahlund K-G, Bergenstahl B, Nilsson L. Macromolecular geometries determined with field-flow fractionation and their impact on the overlap concentration. Biomacromolecules. 2008;9(6):1684–90. 10.1021/bm800127n. 18537296 10.1021/bm800127n

[9] Aberle T, Burchard W, Galinsky G, Hanselmann R, Klingler RW, Michel E. Particularities in the structure of amylopectin, amylose and some of their derivatives in solution. Macromol Symp. 1997;120:47–63. 10.1002/masy.19971200108.

[10] Grubisic Z, Rempp P, Benoit H. A universal calibration for gel permeation chromatography. J Polym Sci Part B Polym Phys. 1996;34(10):1707–14. 10.1002/pol.1967.110050903.

[11] Gaborieau M, Gilbert RG, Gray-Weale A, Hernandez JM, Castignolles P. Theory of multiple detection size exclusion chromatography of complex branched polymers. Macromol Theory Simul. 2007;16(1):13–28. 10.1002/mats.200600046.

[12] Vilaplana F, Gilbert RG. Characterization of branched polysaccharides using multiple-detection size separation techniques. J Separ Sci. 2010;33(22):3537–54. 10.1002/jssc.201000525. 10.1002/jssc.201000525 20960448

[13] Kostanski LK, Keller DM, Hamielec AE. Size-exclusion chromatography - a review of calibration methodologies. Journal of Biochem Biophys Methods. 2004;58(2):159–86. 10.1016/j.jbbm.2003.10.001. 14980789 10.1016/j.jbbm.2003.10.001

[14] Jones RG, Kahovec J, Stepto R, Wilks ES, Hess M, Kitayama T, Metanomski WV. Compendium of polymer terminology and nomenclature. IUPAC Recommendations 2008. Royal Society of Chemistry, Cambridge. 2009. 10.1039/9781847559425

[15] Kuge T, Kobayashi K, Tanahashi H, Igushi T, Kitamura S. Gel permeation chromatography of polysaccharides: universal calibration curve. Agric Biol Chem. 1984;78(9):2375–6. 10.1080/00021369.1984.10866505.

[16] Zhang P, Mazoyer P, Gilbert RG. A broad-standard technique for correcting for band broadening in size-exclusion chromatography. J Chromatography A. 2016;1443(1):267–71. 10.1016/j.chroma.2016.03.030. 10.1016/j.chroma.2016.03.030 27016112

[17] Clay PA, Gilbert RG. Molecular weight distributions in free-radical polymerizations. 1. Model development and implications for data interpretation. Macromolecules. 1995;28:552–69. 10.1021/ma00106a021.

[18] Huber C, Lederer KH. Flow-rate dependent degradation of high-molecular-weight polyisobutylene in GPC. J Polym Sci, Polym Lett Ed. 1980;18(8):535–40. 10.1002/POL.1980.130180804.

[19] Barth HG, Carlin FJ. A review of polymer shear degradation in size-exclusion chromatography. J Liq Chromatograph. 1984;7(9):1717–38. 10.1080/01483918408068832.

[20] Klavons JA, Dintzis FR, Millard MM. Hydrodynamic chromatography of waxy maize starch. Cereal Chem. 1997;74(6):832–6. 10.1094/CCHEM.1997.74.6.832.

[21] Striegel AM. Observations regarding on-column, flow-induced degradation during SEC analysis. J Liquid Chromatograph Relate Technol. 2008;31(20):3105–14. 10.1080/10826070802480024.

[22] Cave RA, Seabrook SA, Gidley MJ, Gilbert RG. Characterization of starch by size-exclusion chromatography: the limitations imposed by shear scission. Biomacromolecules. 2009;10(8):2245–53. 10.1021/bm900426n. 19627139 10.1021/bm900426n

[23] O’Shea MG, Samuel MS, Konik CM, Morell MK. Fluorophore-assisted carbohydrate electrophoresis (FACE) of oligosaccharides - efficiency of labelling and high-resolution separation. Carbohydr Res. 1998;307(1–2):1–12. 10.1016/S0008-6215(97)10085-4.

[24] Wu AC, Morell MK, Gilbert RG. A parameterized model of amylopectin synthesis provides key insights into the synthesis of granular starch. PLoS One. 2013;8(6):e65768. 10.1371/journal.pone.0065768. 23762422 10.1371/journal.pone.0065768 PMC3676345

[25] Nada SS, Zou W, Li C, Gilbert RG. Parameterizing amylose chain-length distributions for biosynthesis-structure-property relations. Anal Bioanalytic Chem. 2017;409(29):6813–9. 10.1007/s00216-017-0639-5. 10.1007/s00216-017-0639-5 28948315

[26] Yu W, Li H, Zou W, Tao K, Zhu J, Gilbert RG. Using starch molecular fine structure to understand biosynthesis-structure-property relations. Trends Food Sci Technol. 2019;86:530–6. 10.1016/j.tifs.2018.08.003.

[27] Zhu J, Gilbert RG. Starch molecular structure and diabetes. Carbohydr Polym. 2024;344:122525. 10.1016/j.carbpol.2024.122525. 39218548 10.1016/j.carbpol.2024.122525

[28] Castro JV, van Berkel KY, Russell GT, Gilbert RG. General solution to the band-broadening problem in polymer molecular weight distributions. Austral J Chem. 2005;58:178–81. 10.1071/CH05002.

[29] Konkolewicz D, Taylor JW II, Castignolles P, Gray-Weale AA, Gilbert RG. Towards a more general solution to the band-broadening problem in size separation of polymers. Macromolecules. 2007;40(9):3477–87. 10.1021/ma062973a.

[30] Wu AC, Gilbert RG. Molecular weight distributions of starch branches reveal genetic constraints on biosynthesis. Biomacromolecules. 2010;11(12):3539–47. 10.1021/bm1010189. 21058715 10.1021/bm1010189

[31] Tao K, Yu W, Gilbert RG. High-amylose rice: molecular structural features controlling cooked rice texture and preference. Carbohydr Polym. 2019;219:251–60. 10.1016/j.carbpol.2019.05.031. 31151523 10.1016/j.carbpol.2019.05.031

[32] Zhu J, Tao K, Prakash S, Zhang C, Gilbert RG, Liu Q. Using starch structure to choose rices with an optimal combination of palatability and digestibility. Food Hydrocol. 2023;141: 108763. 10.1016/j.foodhyd.2023.108763.

[33] Basedow AM, Ebert KH. Ultrasonic degradation of polymers in solution. Adv Polym Sci. 1977;22:83–148. 10.1007/3-540-07942-4_6.

[34] Hanselmann R, Burchard W, Ehrat M, Widmer HM. Structural properties of fractionated starch polymers and their dependence on the dissolution process. Macromolecules. 1996;29(9):3277–82. 10.1021/ma951452c.

[35] van Bruijnsvoort M, Wahlund KG, Nilsson G, Kok WT. Retention behavior of amylopectins in asymmetrical flow field-flow fractionation studied by multi-angle light scattering detection. J Chromatogr A. 2001;925(1–2):171–82. 10.1016/S0021-9673(01)01020-2. 11519803 10.1016/s0021-9673(01)01020-2

[36] Roger P, Baud B, Colonna P. Characterization of starch polysaccharides by flow field-flow fractionation–multi-angle laser light scattering–differential refractometer index. J Chromatography A. 2001;917:179–85. 10.1016/S0021-9673(01)00689-6. 10.1016/s0021-9673(01)00689-6 11403469

[37] You S, Stevenson SG, Izydorczyk MS, Preston KR. Separation and characterization of barley starch polymers by a flow field-flow fractionation technique in combination with multiangle light scattering and differential refractive index detection. Cereal Chem. 2002;79:624–30. 10.1094/CCHEM.2002.79.5.624.

[38] Rolland-Sabate A, Colonna P, Mendez-Montealvo MG, Planchot V. Branching features of amylopectins and glycogen determined by asymmetrical flow field flow fractionation coupled with multiangle laser light scattering. Biomacromolecules. 2007;8(8):2520–32. 17645307 10.1021/bm070024z

[39] Kim W-J, Eum CH, Lim S-T, Han J-H, You S-G, Lee S. Separation of amylose and amylopectin in corn starch using dual-programmed flow field-flow fractionation. Bullet Korean Chem Soc. 2007;28(12):2489–92. 10.5012/bkcs.2007.28.12.2489.

